# Supplementary material for: Multiscale mechanical consequences of ocean acidification for cold-water corals
Source: Sci Rep. 2022 May 16;12:8052. doi: 10.1038/s41598-022-11266-w (PMC9110400; doi:10.1038/s41598-022-11266-w)
Supplement: Supplementary file 1 — Supplementary Information 1. [file 41598_2022_11266_MOESM1_ESM.pdf]

Supplementary material to:

Multiscale Mechanical Consequences of Ocean Acidification for Cold-Water Corals

Uwe Wolfram[1*] Marta Peña Fernández[1] Samuel McPhee[1] Ewan Smith[1] Rainer J. Beck[1] Jonathan D. Shephard[1] Ali Ozel[1] Craig Scott Erskine[1] Janina Büscher[3] Jürgen Titschack[4,5] J Murray Roberts[2] Sebastian Hennige[2]

[1]*School of Engineering and Physical Sciences, Institute of Mechanical, Process and Energy Engineering, Heriot-Watt University, Edinburgh, United Kingdom*
[2]*Changing Oceans Research Group, School of GeoSciences, University of Edinburgh, Edinburgh, United Kingdom*
[3]*GEOMAR Helmholtz Centre for Ocean Research Kiel, Biological Oceanography Research Group, Kiel, Germany*
[4]*Marum Center for Marine Sciences, University of Bremen, Bremen, Germany*
[5]*Senckenberg am Meer, Marine Research Department, Wilhelmshaven, Germany*
[*]*Corresponding author email: u.wolfram@hw.ac.uk*

S1 Electron backscatter diffraction and scanning electron microscopy data

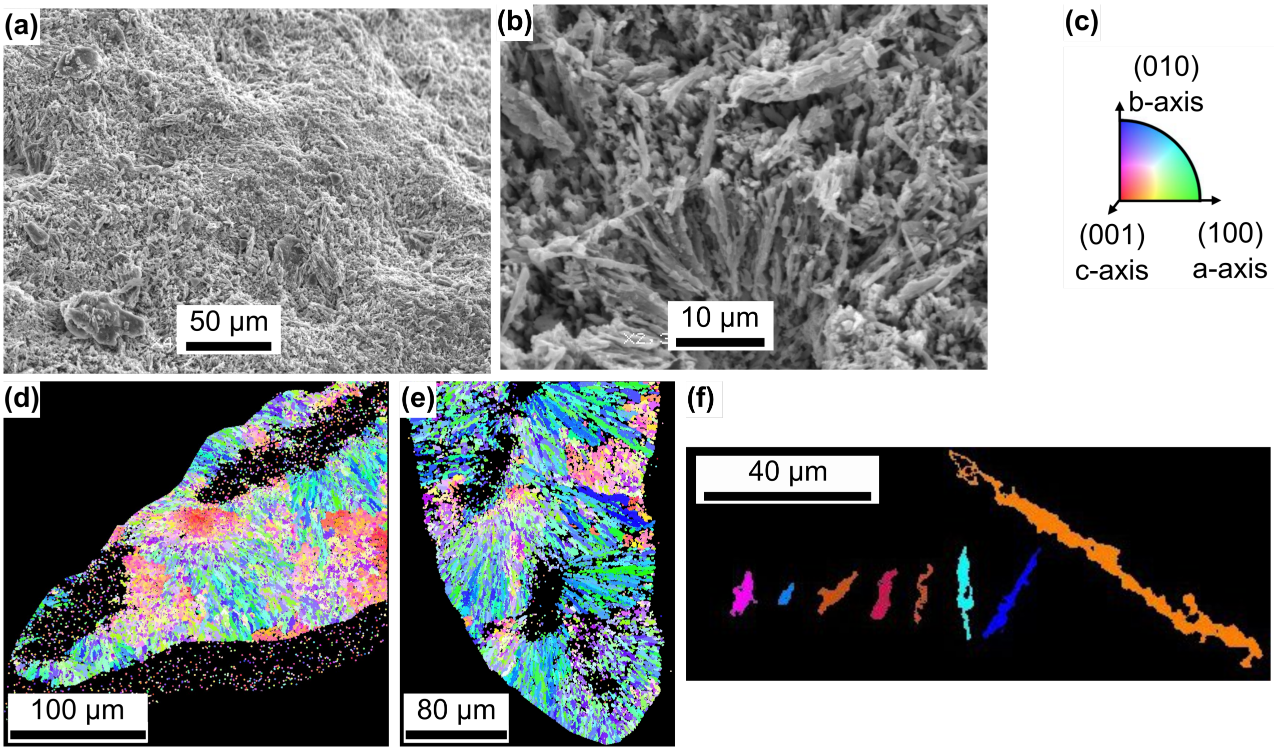

Figure S1 Polycrystalline setup of *L. pertusa*: SEM and EBSD data previously published by Hennige et al. [1] support that aragonite crystals coalesce in a polycrystalline matrix and that a random assembly is a suitable approximation. **(a, b)** are surface SEM images of cold-water coral skeletons. **(c)** shows the crystal coordinate systems used in the inverted pole figure image examples in **(d)** and **(e)** which were obtained using EBSD microscopy. EBSD data **(f)** shows eight examples across the 100 needles analysed which illustrate the range of aspect ratio of 2.01-13.85.

S2 Porosity and affected layer analyses

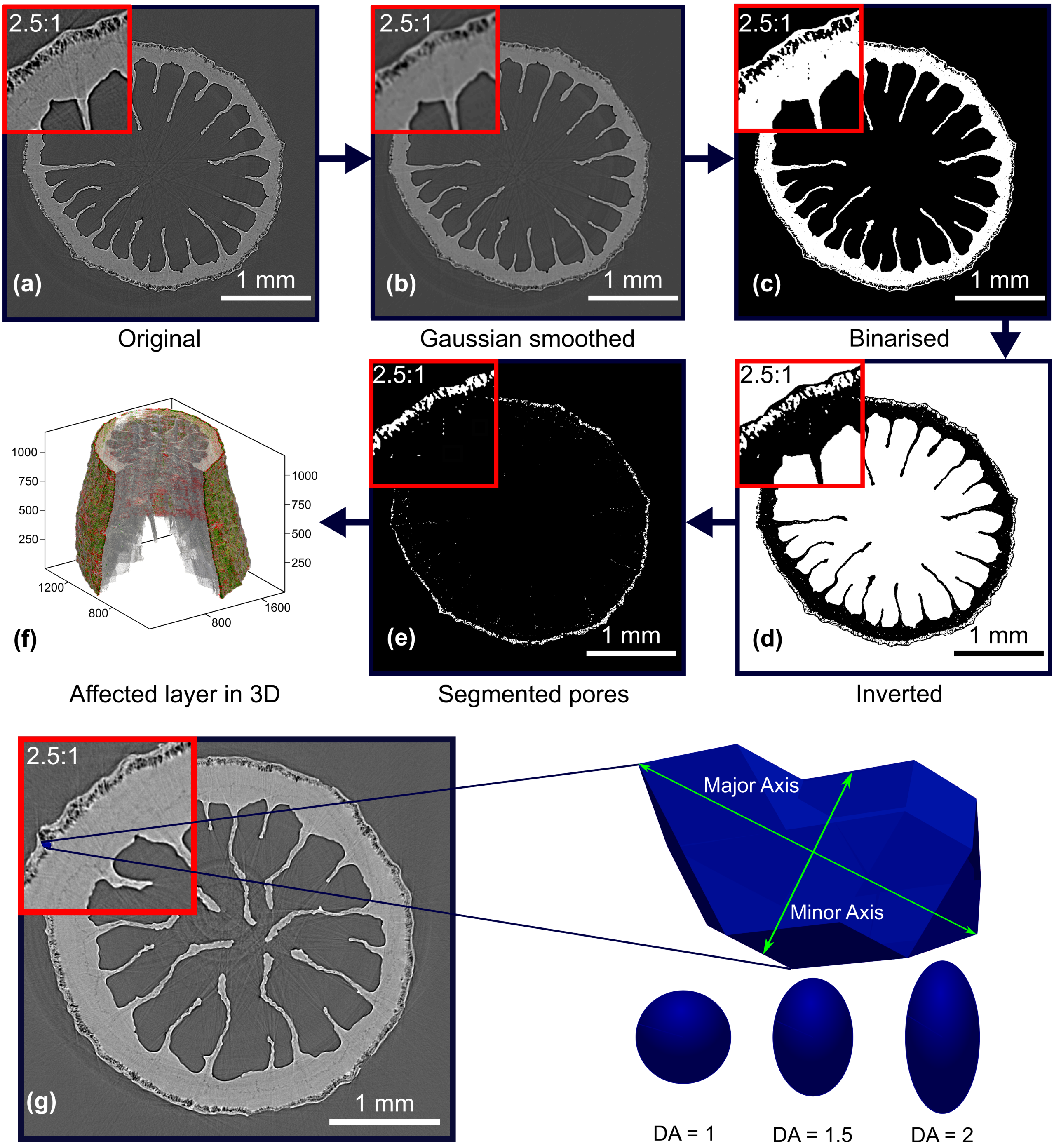

Figure S2: (a-f): Sequential flowchart of porosity analysis. The original image was smoothed and thresholded to produce a binarised image. A mask was then generated using morphological operations (opening and closing). Pixel-wise multiplication of the inverse binarised and mask images enabled extraction of porosity. **(g)** Shape of individual pores were characterised by their Degree of Anisotropy (DA) using a connected component analysis. A larger DA value corresponds to a more oblong shape, while a sphere has a DA = 1.

S3 Eshelby Tensors for Isotropic Inclusions

For an orthogonal material system, the 4th order Eshelby tensor Eshelby [2], $\mathbb{R}$, takes the form,

$$\mathbb{R} = \begin{pmatrix} R_{1111} & R_{1122} & R_{1133} & 0 & 0 & 0 \\ R_{2211} & R_{2222} & R_{2233} & 0 & 0 & 0 \\ R_{3311} & R_{3322} & R_{3333} & 0 & 0 & 0 \\ 0 & 0 & 0 & 2R_{2323} & 0 & 0 \\ 0 & 0 & 0 & 0 & 2R_{1313} & 0 \\ 0 & 0 & 0 & 0 & 0 & 2R_{1212} \end{pmatrix} \qquad (S1)$$

The individual components of the Eshelby tensor for inclusion shapes used in this paper are provided below (solutions obtained from Mura [3] and David and Zimmerman [4]) where,

$\nu =$ Poisson's ratio of isotropic matrix

$a =$ Inclusion aspect ratio

$\mathbb{R}_{asi}$ - Aligned spheroidal inclusions

$$\mathbb{R}_{1111} = \mathbb{R}_{2222} = -\frac{3a^2}{8(1-\nu)(1-a^2)} + \frac{1}{4(1-\nu)}\left[1 - 2\nu + \frac{9}{4(1-a^2)}\right] g \qquad (S2)$$

$$\mathbb{R}_{3333} = \frac{1}{1-\nu}\left[2 - \nu + \frac{1}{1-a^2}\right] + \frac{1}{2(1-\nu)}\left[-2(2-\nu) + \frac{3}{1-a^2}\right] g \qquad (S3)$$

$$\mathbb{R}_{1122} = \mathbb{R}_{2211} = \frac{1}{8(1-\nu)}\left[1 - \frac{1}{1-a^2}\right] + \frac{1}{16(1-\nu)}\left[-4(1-2\nu) + \frac{3}{1-a^2}\right] g \qquad (S4)$$

$$\mathbb{R}_{2233} = \mathbb{R}_{1133} = \frac{a^2}{2(1-\nu)(1-a^2)} + \frac{1}{4(1-\nu)}\left[1 - 2\nu + \frac{3a^2}{1-a^2}\right] g \qquad (S5)$$

$$\mathbb{R}_{3311} = \mathbb{R}_{3322} = \frac{1}{2(1-\nu)}\left[-(1-2\nu) + \frac{1}{1-a^2}\right] + \frac{1}{4(1-\nu)}\left[2(1-2\nu) - \frac{3}{1-a^2}\right] g \qquad (S6)$$

$$\mathbb{R}_{1212} = \mathbb{R}_{2121} = \frac{a^2}{8(1-\nu)(1-a^2)} + \frac{1}{16(1-\nu)}\left[4(1-2\nu) + \frac{3}{1-a^2}\right] g \qquad (S7)$$

$$\mathbb{R}_{1313} = \mathbb{R}_{2323} = \frac{1}{4(1-\nu)}\left[1 - 2\nu + \frac{1+a^2}{1-a^2}\right] - \frac{1}{8(1-\nu)}\left[1 - 2\nu + 3\frac{1+a^2}{1-a^2}\right] g \qquad (S8)$$

where g is a function of the inclusion aspect ratio, a, and takes two forms depending on whether the inclusions are prolate spheroids ($a > 1$) or oblate spheroids ($a < 1$):

$$g = \frac{a}{(a^2-1)^{\frac{3}{2}}}\left(a(a^2-1)^{\frac{1}{2}} - \cosh^{-1} a\right) \qquad \text{when } a > 1 \qquad (S9)$$

$$g = \frac{a}{(1-a^2)^{\frac{3}{2}}}\left(\cos^{-1} a - a(1-a^2)^{\frac{1}{2}}\right) \qquad \text{when } a < 1 \qquad (S10)$$

$\mathbb{R}_{rsi}$ - Random spherical inclusions $(a_1 = a_2 = a_3 = a)$

$$\mathbb{R}_{1111} = \mathbb{R}_{2222} = \mathbb{R}_{3333} = \frac{7-5\nu}{15(1-\nu)} \qquad (S11)$$

$$\mathbb{R}_{1212} = \mathbb{R}_{2323} = \mathbb{R}_{3131} = \frac{4-5\nu}{15(1-\nu)} \qquad (S12)$$

$$\mathbb{R}_{1122} = \mathbb{R}_{2211} = \mathbb{R}_{3311} = \mathbb{R}_{1133} = \mathbb{R}_{2211} = \mathbb{R}_{3322} = \frac{5\nu-1}{15(1-\nu)} \qquad (S13)$$

S4 Aragonite needle orientation

To find the most adversely loaded aragonite needle we employ needle coordinate systems introduced for randomly oriented hydroxyapatite needles[5, 6]:

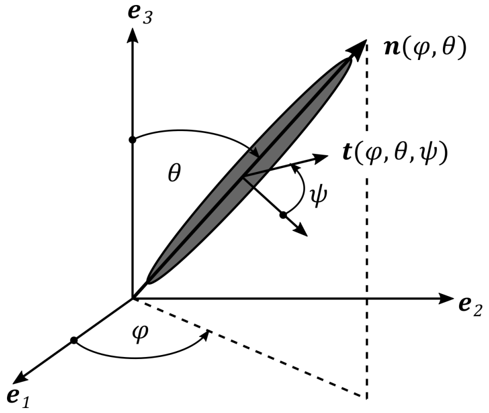

Figure S3: Crystal orientation characterised by Euler angles φ, θ and tangential vector $\boldsymbol{t}$ characterised by Euler angles φ, θ and the in-plane rotation ψ.

S5 Micropillar compression

Two arrays of six micropillars were positioned on the skeletal wall of a CWC sample. Figure S4 illustrates micropillar location and shape on a representative sample.

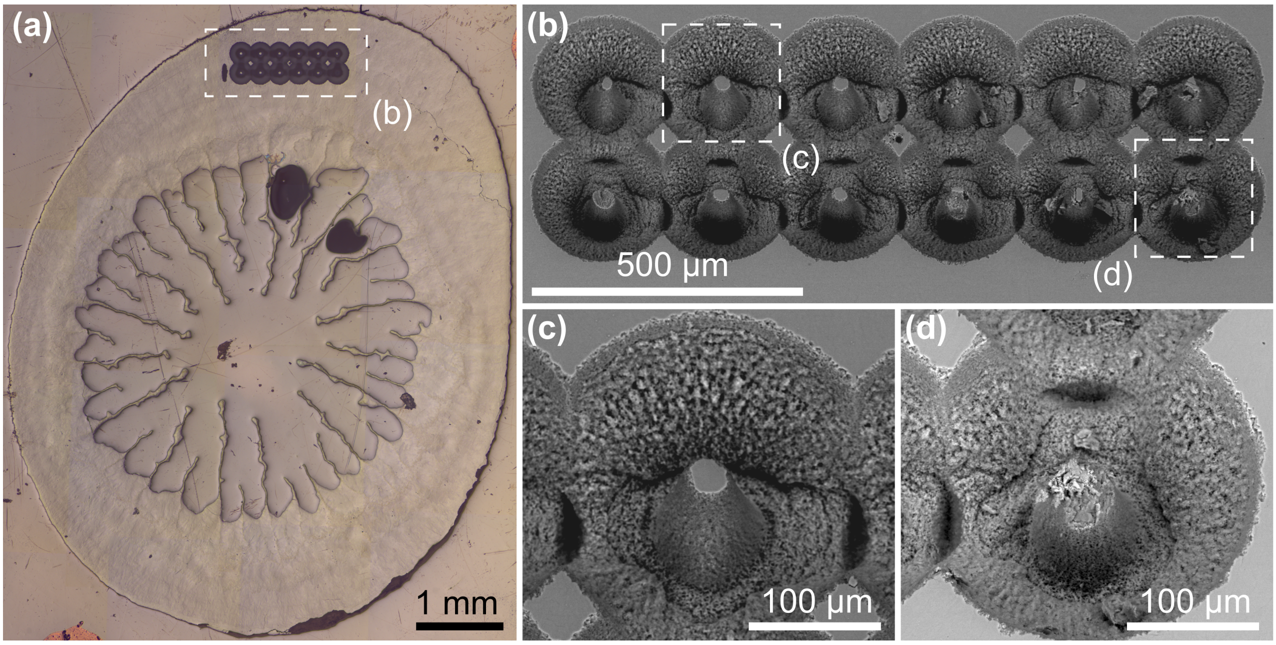

Figure S4 Coral micropillars: (a) reflected light microscopy image of a corallite cross section. **(b)** SEM image of a laser ablated array of 2x6 micropillars with the left six pillars before and the right six pillars after testing. **(c)** SEM of an untested micropillar and **(d)** SEM of a tested micropillar.

We fabricated micropillars using an ultrashort pulsed laser machining workstation based on a Carbide laser (Light Conversion) with a laser wavelength of $\lambda = 1028$ nm, a pulse length of 6 ps, and a repetition rate of 2 kHz. We used a focussed spot size of 20 µm, 89% beam overlap, and 11% spot separation. The laser was operated at a pulse energy of 10.3 µJ and the beam was brought to

focus into a 20 µm diameter laser spot positioned 70 µm below the top surface of the coral tissue. The focussed laser beam was scanned across the sample in an inward Archimedean spiral pattern with a speed of 4.4 mm/s by means of a galvanometer scan head. The outer diameter of the spiral was 250 µm and the internal diameter 44 µm. This scanning pattern was repeated three times to create 2 x 6 pillar grids on each sample. After laser ablation, samples were cleaned in an ultrasound bath for 5 s to displace any material ejecta from the coral surface and then glued to SEM stubs for mounting.

S6 Obtaining cohesion h and friction coefficient T

The equations for $\mathbb{F}$ and $\mathbf{F}$ in equation (9) of the main manuscript are a porous version of a generalised Drucker-Prager criterion provided by Schwiedrzik et al. [7] with tensors:

$$\mathbb{F} = \frac{3}{2} F_0^2 \boldsymbol{I} \,\overline{\underline{\otimes}}\, \boldsymbol{I} - \frac{1}{2} F_0^2 \boldsymbol{I} \otimes \boldsymbol{I} \text{ and } \boldsymbol{F} = f_0 \boldsymbol{I} \text{ with } F_0 = \frac{1}{2} \frac{\sigma^{uc} + \sigma^{ut}}{\sigma^{uc}\sigma^{ut}} \text{ and } f_0 = \frac{1}{2} \frac{\sigma^{uc} - \sigma^{ut}}{\sigma^{uc}\sigma^{ut}}. \quad (S14)$$

Comparing (S1) with (9) for $\phi_{OA} = 0$ allows us to deduce T and h as:

$$T = \sqrt{6} \frac{f_0}{F_0} = \sqrt{6} \frac{\sigma^{uc} - \sigma^{ut}}{\sigma^{uc} + \sigma^{ut}} \quad \text{and} \quad h = \frac{1}{3 f_0} = \frac{2}{3} \frac{\sigma^{uc}\sigma^{ut}}{\sigma^{uc} - \sigma^{ut}}. \quad (S15)$$

S7 Elasto-viscoplastic material model and its implementation

We implemented this micromechanical model as a UMAT in Abaqus (6.16, Dassault Systèmes) following a similar material model for bone tissue[8]. We propose a linear elastic-viscoplastic material model whose elasticity is governed by our three-step micromechanical scheme (Figure 2). The elastic domain is limited by the Drucker-Prager type yield surface for the skeleton exposed to corrosive waters (Section 2.8). Motivated by 0.1-5wt% organic matter incorporated in the skeletal matrix as well as the creep behaviour in our nanoindentation experiments[9], we propose a viscoplastic post-yield behaviour (Figure S5). This allows us to adopt the model developed by Schwiedrzik and Zysset[8] for bone tissue. We present the governing equations since there are significant differences compared to Schwiedrzik and Zysset[8]. The observed mechanical behaviour shows no damage and features multiscale elasticity and a micromechanical yield surface with very short post-yield region under compression.

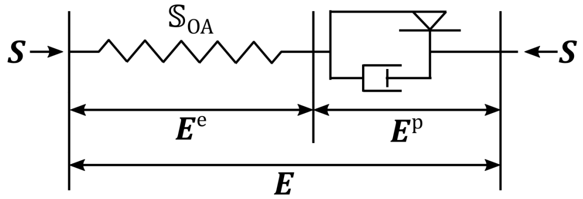

Figure S5 Rheological model: We propose a material model that consists of a linear-elastic spring and a short post-yield behaviour that is governed by a frictional slider and a dashpot to model viscoplastic behaviour. Viscosity here is motivated by 0.1-5wt% organic matter incorporated in the skeletal matrix.

We assume small deformations so that a Green-Naghdi split of total strain $\boldsymbol{E}$ into its elastic and plastic part is usable[10] and the accumulated plastic strain κ can be defined as:

$$\boldsymbol{E} = \boldsymbol{E}^e + \boldsymbol{E}^p$$
$$\kappa = \int_0^t \left|\left|\dot{\boldsymbol{E}}^p\right|\right| d\tau\,. \qquad (S16)$$

Using this, the free energy potential for this material takes on the form:

$$2\psi(\boldsymbol{E}, \boldsymbol{E}^p) = (\boldsymbol{E} - \boldsymbol{E}^p) : \mathbb{S}_{OA}(\boldsymbol{E} - \boldsymbol{E}^p) \qquad (S17)$$

with $\mathbb{S}_{OA}$ the fourth order stiffness tensor for the acidified skeleton introduced in equation (8) of the main manuscript. Consequently, the stress is derived as

$$\boldsymbol{S} = \nabla_{\boldsymbol{E}}\psi(\boldsymbol{E}, \boldsymbol{E}^p) = \mathbb{S}_{OA} : (\boldsymbol{E} - \boldsymbol{E}^p)$$
$$\boldsymbol{S}^p = -\nabla_{\boldsymbol{E}^p}\psi(\boldsymbol{E}, \boldsymbol{E}^p) = \mathbb{S}_{OA} : (\boldsymbol{E} - \boldsymbol{E}^p) \qquad (S18)$$

and the dissipation becomes

$$\Phi = \boldsymbol{S} : \dot{\boldsymbol{E}} - \dot{\psi} \geq 0$$
$$\Phi = \boldsymbol{S} : \dot{\boldsymbol{E}} - \boldsymbol{S} : \dot{\boldsymbol{E}} + \boldsymbol{S} : \dot{\boldsymbol{E}}^p = \boldsymbol{S} : \dot{\boldsymbol{E}}^p \geq 0. \qquad (S19)$$

Motivated by 0.1-5wt% organic matter incorporated in the skeletal matrix as well as mild creep encountered in our nanoindentation experiments[9] we implement a viscoplasticity as proposed by Perzyna[8, 11, 12]:

$$\dot{\boldsymbol{E}}^p = \frac{1}{\eta}\langle\chi(Y_{OA})\rangle\boldsymbol{M}^p$$
$$\boldsymbol{M}^p = \nabla_{\boldsymbol{S}} Y_{OA}. \qquad (S20)$$

$\langle \dots \rangle$ represent the McAuley brackets, Y_{OA} is the yield surface introduced in equation (9), and $\chi(Y_{OA})$ is a monotonously increasing and invertible overstress function necessary for Perzyna-type materials[8]. As proposed by Schwiedrzik and Zysset [8], we implemented a continuous Perzyna viscoplasticity through introducing a viscoplastic consistency parameter $\dot{\lambda}$ that we will substitute into the flow rule:

$$\dot{\lambda} = \frac{1}{\eta}\langle\chi(Y_{OA})\rangle$$
$$\dot{\boldsymbol{E}}^p = \frac{1}{\eta}\dot{\lambda}\boldsymbol{M}^p \qquad (S21)$$

We constrain the viscoplastic flow by exploiting invertibility of the overstress function so that we are able to formulate generalised Karush-Kuhn-Tucker conditions[8]:

$$Y_{OA} = \chi^{-1}(\dot{\lambda}\eta)$$
$$\bar{Y}_{OA} = Y_{OA} - \chi^{-1}(\dot{\lambda}\eta) = 0 \qquad (S22)$$
$$\bar{Y}_{OA} \leq 0, \quad \dot{\lambda} \geq 0, \quad \dot{\lambda}\bar{Y}_{OA} = 0$$

This allows us then to implement the model as a UMAT in Abaqus (v6.16, Dassault Systémes). In the following, the increment number n is omitted and converged variables A_{n+1} will be called A while variables at the beginning of the increment will be called A_0. The increment number is different from the local iteration number i of the Newton–Raphson algorithm which we use to compute the unknowns $\boldsymbol{E}^p$ and κ. We start the computation with identifying a trial stress:

$$\boldsymbol{S}^T = \mathbb{S}_{OA}:\left(\boldsymbol{E} - \boldsymbol{E}_0^p\right) \tag{S23}$$

If the yield criterion is not violated by using this stress, i.e. $Y_{OA}(\boldsymbol{S}^T;\kappa_0) < 0$, the stress increment is elastic and the state variables can be updated as

$$\begin{gathered} \kappa = \kappa_0 \\ \boldsymbol{E}^p = \boldsymbol{E}_0^p \\ \boldsymbol{S} = \boldsymbol{S}^T \end{gathered} \tag{S24}$$

and the tangent stiffness tensor is simply the elastic stiffness tensor $\mathbb{S}_{OA}$.

If $Y_{OA}(\boldsymbol{S}^T;\kappa_0) \geq 0$, an implicit stress return algorithm[13] is carried out respecting the Karush-Kuhn-Tucker conditions (S22.3) and the set of nonlinear equations to be solved is:

$$\begin{gathered} \boldsymbol{S} = \mathbb{S}_{OA}:(\boldsymbol{E} - \boldsymbol{E}^p) \\ \bar{Y}_{OA}\left(\boldsymbol{S},\kappa,\dot{\lambda}\right) = Y_{OA}(\boldsymbol{S},\kappa) - \chi^{-1}\left(\dot{\lambda},\eta\right) = 0 \\ \dot{\boldsymbol{E}^p} = \dot{\lambda}\nabla_{\mathbf{S}}Y_{OA}(\boldsymbol{S},\kappa) \\ \dot{\kappa} = \left|\left|\dot{\boldsymbol{E}^p}\right|\right| = \dot{\lambda}\left|\left|\nabla_{\mathbf{S}}Y_{OA}(\boldsymbol{S},\kappa)\right|\right|. \end{gathered} \tag{S25}$$

The incremental Lagrangean multiplier $\Delta\lambda$ can be written as a function of the incremental accumulated plastic strain $\Delta\lambda = \frac{\Delta\kappa}{||\nabla_{\mathbf{S}}Y_{OA}(\boldsymbol{S},\kappa_0+\Delta\kappa)||}$ so that $\Delta\boldsymbol{E}^p = \Delta\kappa\frac{\nabla_{\mathbf{S}}Y_{OA}(\boldsymbol{S},\kappa)}{||\nabla_{\mathbf{S}}Y_{OA}(\boldsymbol{S},\kappa_0+\Delta\kappa)||} = \Delta\kappa\boldsymbol{N}^p$. The polynomial flow rule for $\dot{\lambda}$ and the overstress function χ can be specified[8] as:

$$\begin{gathered} \dot{\lambda} = \frac{1}{\eta}\left(Y_{OA}(\boldsymbol{S},\kappa)^2 + mY_{OA}(\boldsymbol{S},\kappa)\right) \\ \chi^{-1}\left(\dot{\lambda},\eta\right) = -\frac{m}{2} + \sqrt{\frac{m^2}{4} + \eta\dot{\lambda}} \end{gathered} \tag{S26}$$

with $m = 1$ and $\eta = 1 \cdot 10^{-4}$ MPa·s.

Total strain at the end of the load increment is given and can be separated into $\boldsymbol{E} = \boldsymbol{E}_0 + \Delta\boldsymbol{E}$. With this the stress can be given in incremental form:

$$\boldsymbol{S} = \mathbb{S}_{OA}:\left(\boldsymbol{E}_0 + \Delta\boldsymbol{E} - \boldsymbol{E}_0^p - \Delta\boldsymbol{E}^p\right) = \boldsymbol{S}^T - \mathbb{S}_{OA}\Delta\boldsymbol{E}^p = \boldsymbol{S}^T - \mathbb{S}_{OA}\Delta\kappa\boldsymbol{N}^p \tag{S27}$$

wherein $\boldsymbol{S}^T = \mathbb{S}_{OA}:\left(\boldsymbol{E} - \boldsymbol{E}_0^p\right)$. We now multiply from the left with $\mathbb{E}_{OA} = \mathbb{S}_{OA}^{-1}$ and bring everything to one side to derive the residual error of the elastic strains

$$\boldsymbol{R}(\boldsymbol{S}, \Delta\kappa) = \mathbb{E}_{OA}(\boldsymbol{S} - \boldsymbol{S}^T) + \Delta\kappa \boldsymbol{N}^p. \tag{S28}$$

Similarly, we approximate the rate-dependent yield function by

$$\bar{Y}_{OA}(\boldsymbol{S}, \Delta\kappa) = Y_{OA}(\boldsymbol{S}, \kappa_0 + \Delta\kappa) - \chi^{-1}\left(\frac{\Delta\kappa}{\left|\left|\nabla_{\boldsymbol{S}} Y_{OA}(\boldsymbol{S}, \kappa_0 + \Delta\kappa)\right|\right|} \frac{1}{\Delta t}, \eta\right). \tag{S29}$$

(S28) and (S29) are linearised around the solution with respect to the variables $\boldsymbol{S}$ and $\Delta\kappa$ so that a linearised system of equations can be established:

$$\begin{aligned} \boldsymbol{R}^{i+1} &= \boldsymbol{R}^i + \nabla_{\boldsymbol{S}} \boldsymbol{R}^i : \delta\boldsymbol{S} + \frac{\partial \boldsymbol{R}^i}{\Delta\kappa^i} \delta\Delta\kappa = \boldsymbol{0} \\ \bar{Y}_{OA}^{i+1} &= \bar{Y}_{OA}^i + \nabla_{\boldsymbol{S}} \bar{Y}_{OA}^i \delta\boldsymbol{S} + \frac{\partial \bar{Y}_{OA}^i}{\Delta\kappa^i} \delta\Delta\kappa = 0 \end{aligned} \tag{S30}$$

This is then solved iteratively using $\mathbb{S}_a = -(\nabla_{\boldsymbol{S}} \boldsymbol{R})^{-1}$ and

$$\delta\Delta\kappa = -\frac{\frac{\bar{Y}_{OA}^i}{\left|\left|\nabla_S \bar{Y}_{OA}^i\right|\right|} + \boldsymbol{N}^p \mathbb{S}_a \boldsymbol{R}^i}{\boldsymbol{N}^p \mathbb{S}_a \frac{\partial \boldsymbol{R}^i}{\partial \Delta\kappa^i} + \frac{\frac{\partial \bar{Y}_{OA}^i}{\partial \Delta\kappa^i}}{\left|\left|\nabla_S \bar{Y}_{OA}^i\right|\right|}} \qquad \text{and} \qquad \delta\boldsymbol{S} = \mathbb{S}_a\left(\boldsymbol{R}^i + \frac{\partial \boldsymbol{R}^i}{\Delta\kappa^i} \delta\Delta\kappa\right) \tag{S31}$$

until $\left|\left|\boldsymbol{R}\right|\right| < tol$ and $Y < tol$ wherein tol is a predefined tolerance. The solution in each iteration is used to update the current state of the variables:

$$\begin{aligned} \boldsymbol{S}^{i+1} &= \boldsymbol{S}^i + \delta\boldsymbol{S} \\ \Delta\kappa^{i+1} &= \Delta\kappa^i + \delta\Delta\kappa \end{aligned} \tag{S32}$$

Once converged, the state variables can be updated so that $\boldsymbol{S} = \boldsymbol{S}^{i+1}$, $\kappa = \kappa_0 + \Delta\kappa^{i+1}$, and $\boldsymbol{E}^p = \boldsymbol{E} - \mathbb{E}_{OA}\boldsymbol{S}$.

According to Simo and Hughes [13] the algorithmic tangent tensor $\mathbb{S}_{CA}$ can be found through a linearisation of the stress-strain relationship around the current solution and by enforcing consistency. Schwiedrzik and Zysset [8] conclude that this is already done during application of the Newton-Raphson scheme for stress integration. Therefore, the tensor relating infinitesimal changes in strain $\delta\boldsymbol{R}$ to infinitesimal changes in stress $\delta\boldsymbol{S}$ (i.e. the tangent operator) can be found by substituting (S31.1) at $Y_{OA} = 0$ into (S31.2):

$$\delta\boldsymbol{S} = \mathbb{S}_a \boldsymbol{R}^i - \mathbb{S}_a \frac{\partial \boldsymbol{R}^i}{\Delta\kappa^i}\left(\frac{\boldsymbol{N}^p \mathbb{S}_a \boldsymbol{R}^i}{\boldsymbol{N}^p \mathbb{S}_a \frac{\partial \boldsymbol{R}^i}{\partial \Delta\kappa^i} + \frac{\frac{\partial \bar{Y}_{OA}^i}{\partial \Delta\kappa^i}}{\left|\left|\nabla_S \bar{Y}_{OA}^i\right|\right|}}\right) = \left(\mathbb{S}_a - \frac{\mathbb{S}_a\left(\frac{\partial \boldsymbol{R}^i}{\partial \Delta\kappa^i} \otimes \boldsymbol{N}^p\right)\mathbb{S}_a}{\boldsymbol{N}^p \mathbb{S}_a \frac{\partial \boldsymbol{R}^i}{\partial \Delta\kappa^i} + \frac{\frac{\partial \bar{Y}_{OA}^i}{\partial \Delta\kappa^i}}{\left|\left|\nabla_S \bar{Y}_{OA}^i\right|\right|}}\right)\boldsymbol{R}^i \tag{S33}$$

$$\delta\boldsymbol{S} = \mathbb{S}_{CA}\boldsymbol{R}^i$$

The derivatives used in (S30) to (S33) amount to:

$$\nabla_{\boldsymbol{S}} \boldsymbol{R}^i = \mathbb{E}_{OA} + \Delta\kappa^i \nabla_{\boldsymbol{S}} \boldsymbol{N}^p$$
$$\frac{\partial \boldsymbol{R}^i}{\partial \Delta\kappa^i} = \boldsymbol{N}^p + \Delta\kappa^i \frac{\partial \boldsymbol{N}^p}{\partial \Delta\kappa^i} \qquad (S34)$$

$$\nabla_{\boldsymbol{S}} \boldsymbol{N}^p = \frac{\nabla_{\boldsymbol{S}}\left(\nabla_{\boldsymbol{S}} Y_{OA}^i\right) \left\|\nabla_{\boldsymbol{S}} Y_{OA}^i\right\| - \nabla_{\boldsymbol{S}} Y_{OA}^i \otimes \left(\nabla_{\boldsymbol{S}} \left\|\nabla_{\boldsymbol{S}} Y_{OA}^i\right\|\right)}{\left\|\nabla_{\boldsymbol{S}} Y_{OA}^i\right\|^2}. \qquad (S35)$$

For the rate independent yield surface, we introduce a hardening function $r(\kappa)$ that allows to take different forms of post-yield behaviour such as exponential hardening or softening into account so that the yield surface and associated derivatives become:

$$Y_{OA}\left(\boldsymbol{S}, \Delta\kappa^i\right) := \sqrt{\boldsymbol{S}: \mathbb{F}\boldsymbol{S}} + \boldsymbol{F}: \boldsymbol{S} - r\left(\kappa_0 + \Delta\kappa^i\right) = 0 \qquad (S36)$$

$$\frac{\partial Y_{OA}^i}{\partial \Delta\kappa^i} = -r'\left(\Delta\kappa^i\right) \quad \text{and} \quad \nabla_{\boldsymbol{S}} Y_{OA}^i = \frac{\mathbb{F}: \boldsymbol{S}}{\sqrt{\boldsymbol{S}: \mathbb{F}\boldsymbol{S}}} + \boldsymbol{F} \qquad (S37)$$

$$\frac{\partial \left\|\nabla_{\boldsymbol{S}} Y_{OA}^i\right\|}{\partial \Delta\kappa^i} = 0 \quad \text{and} \quad \frac{\partial \nabla_{\boldsymbol{S}} Y_{OA}^i}{\partial \Delta\kappa^i} = 0 \qquad (S38)$$

$$\nabla_{\boldsymbol{S}}\left(\nabla_{\boldsymbol{S}} Y_{OA}^i\right) = \frac{\mathbb{F}(\boldsymbol{S}: \mathbb{F}\boldsymbol{S}) - (\boldsymbol{S}: \mathbb{F})^T \otimes (\mathbb{F}: \boldsymbol{S})}{(\boldsymbol{S}: \mathbb{F}\boldsymbol{S})^{\frac{3}{2}}}$$
$$\nabla_{\boldsymbol{S}} \left\|\nabla_{\boldsymbol{S}} Y_{OA}^i\right\| = \frac{\nabla_{\boldsymbol{S}}\left(\nabla_{\boldsymbol{S}} Y_{OA}^i\right): \nabla_{\boldsymbol{S}} Y_{OA}^i + \nabla_{\boldsymbol{S}} Y_{OA}^i: \left(\nabla_{\boldsymbol{S}} Y_{OA}^i\right)}{\left\|\nabla_{\boldsymbol{S}} Y_{OA}^i\right\|}. \qquad (S39)$$

The derivatives for the rate dependent yield surface are then a combination of the rate independent case and the viscous correction:

$$\bar{Y}_{OA}(\boldsymbol{S}, \Delta\kappa) = Y_{OA}\left(\boldsymbol{S}, \kappa_0 + \Delta\kappa^i\right) + \frac{m}{2} - \sqrt{\frac{m^2}{4} + \frac{\eta}{\Delta t} \frac{\Delta\kappa^i}{\left\|\nabla_{\boldsymbol{S}} Y_{OA}\left(\boldsymbol{S}, \kappa_0 + \Delta\kappa^i\right)\right\|}} \qquad (S40)$$

$$\bar{Y}_{OA}^i = Y_{OA}^i + \frac{m}{2} - \left(\frac{m^2}{4} + \frac{\eta}{\Delta t} \frac{\Delta\kappa^i}{\left\|\nabla_{\boldsymbol{S}} Y_{OA}^i\right\|}\right)^{\frac{1}{2}}$$
$$\nabla_{\boldsymbol{S}} \bar{Y}_{OA}^i = \nabla_{\boldsymbol{S}} Y_{OA}^i + \frac{1}{2}\left(\frac{m^2}{4} + \frac{\eta}{\Delta t} \frac{\Delta\kappa^i}{\left\|\nabla_{\boldsymbol{S}} Y_{OA}^i\right\|}\right)^{-\frac{1}{2}} \frac{\eta}{\Delta t} \frac{\nabla_{\boldsymbol{S}} \left\|\nabla_{\boldsymbol{S}} Y_{OA}^i\right\|}{\left\|\nabla_{\boldsymbol{S}} Y_{OA}^i\right\|^2} \Delta\kappa^i \qquad (S41)$$
$$\frac{\partial \bar{Y}_{OA}^i}{\partial \Delta\kappa^i} = \frac{\partial Y_{OA}^i}{\partial \Delta\kappa^i} + \frac{1}{2}\left(\frac{m^2}{4} + \frac{\eta\Delta\kappa^i}{\Delta t \left\|\nabla_{\boldsymbol{S}} Y_{OA}^i\right\|}\right)^{-\frac{1}{2}} \frac{\eta}{\Delta t} \frac{1}{\left\|\nabla_{\boldsymbol{S}} Y_{OA}^i\right\|}$$

This material model was used to interpret the micropillar compression tests (Sections 3.7, 3.4) and to simulate CWC deformation due to a distributed pressure (Section 3.5, Figure S6).

S7.1 Interpreting micropillar compression tests

To interpret the micropillar compression tests, pillar dimensions were based upon the median dimensions of the manufactured micropillars (Sections 2.7 and 3.4) with a top radius of 14.48 μm, 43.17 μm radius for the base, and 110.77 μm pillar height. Substrate material was modelled with a radius and length of 150 μm to accommodate "sink-in" of the pillar as it is compressed. One quarter of the pillar and substrate was modelled with symmetry constraints in the x-z and y-z planes applied accordingly (Figure S6). The bottom surface of the substate was constrained against displacement in the loading testing direction (y-axis). The microindenter was not modelled but represented through a displacement boundary condition applied to the top surface. Displacement was set to 3 μm, enough to pass the yield point of the material. The entire model was meshed with quadratic tetrahedral elements (C3D10, Figure S6). Due to the complexity of the stress field under the pillar base, meshing was constrained by a single bias seed to maintain a fine mesh, while the substrate's mesh seed is biased to reduce in size as it approaches the edges of the model to save element count and thus computation cost. A mesh sensitivity analyses was conducted to evaluate at which point the recorded reaction force saturates and does not change with further mesh refinement. For this pillar this was found to be at 454,786 elements. Force-displacement data was retreived from the model by recording the displacement of the pillar's top surface and the cummulative reaction force upon every node on the substrate base.

S7.2 Simulating CWC deformation due to a distributed pressure

Surface and volumetric meshes (Section 2.9) were imported into Matlab (R2020a), where input files for Abaqus (v6.16, Dassault Systémes) were generated. The coral skeleton surface was subjected to a constant pressure load simulating an arbitrarily chosen sea current with a velocity of 3 m/s in a direction perpendicular to the longitudinal axis (z-axis) of the skeleton (Figure S6). This velocity was chosen to firmly overload the sample and represents three times the maximum water current reported by Haugan et al. [14] (0.3-1 m/s). It is important to note that this is an academic example to illustrate the effect of increasing velocity and loss of skeletal wall thickness. First, the normal of all external faces were computed and those facing the flow direction identified. A ray-triangle intersection algorithm[15] was then used to calculate the intersection of a ray in the direction of the flow and the triangulated surface mesh. This allowed us to detect those faces not shielded by other elements, thus, isolating the triangular faces on the coral skeleton surface where the rays first impinge on. The tetrahedral elements to which such faces belonged were identified and a distributed surface pressure p was applied following $p = {}^{1}\!/_{2}\, \rho v^2 \cos \alpha$. ρ is the density of the sea water ($\rho = 1026$ kg m^{-3}), v the velocity of the current, and α the angle between the direction of the sea current flow and the normal

of the faces hit by the flow (Figure S6). We omitted shear forces and the hydrostatic pressure for simplicity knowing that they would contribute to the actual mechanical situation. Considering flow in our example was sufficient to illustrate the impact of ocean acidification on load bearing capacity. Finally, linear tetrahedral elements were converted to quadratic ones and all degrees of freedom in the nodes of the most distal portion of the skeleton (10 mm height) were constrained to simulate a coral fixed at its root.

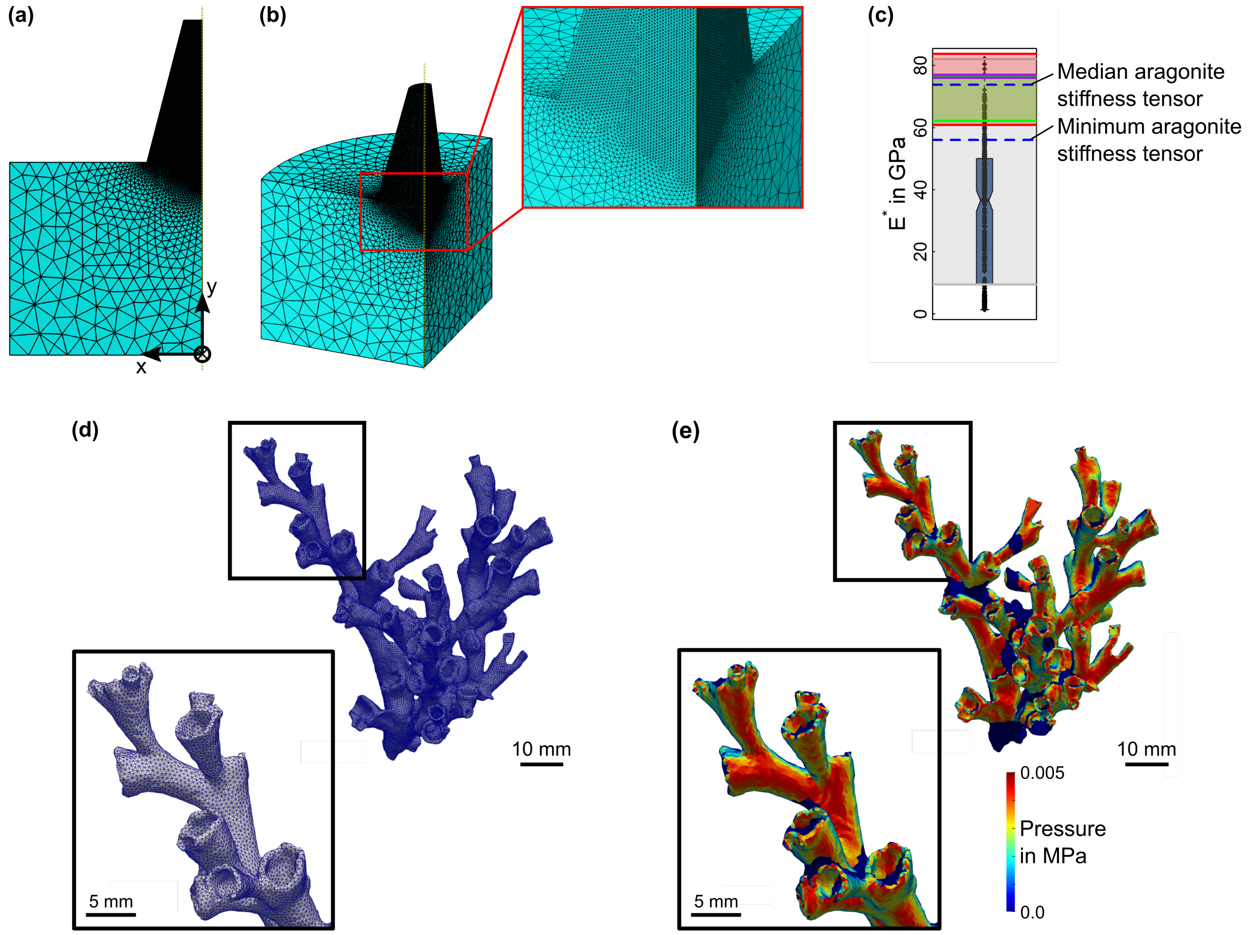

Figure S6 Finite element (FE) models used in the study: (a) and **(b)** finite element mesh of the fabricated micropillars to identify compressive strength of the polycrystalline material (Sections 2.7 and 3.4). **(c)** Stiffness obtained by nanoindentation tests (Section S8). The areas represent ranges for stiffness found in the literature (grey: California Sea Bight[9], red: shallow water corals around volcanic sites in the Mediterranean Sea[16], green: non-zooxanthellate and zooxanthellate corals from the Mediterranean Sea[17]; purple: scleractinian corals from Mediterranean and tropical waters[18]) The dashed blue lines **(c)** illustrate the results from our modelling (Section 3.3). Our model prediction using median aragonite stiffness fits very well to results by Pasquini et al. [18]. **(d)** Tetrahedral mesh of a representative coral sample that was used to investigate the impact of ocean acidification. **(e)** pressure boundary conditions loading the coral with a distributed flow pressure.

S8 Measuring skeletal stiffness using nanoindentation

Hennige et al. [9] measured skeletal stiffnesses of *L. pertusa* samples from the California Sea Bight which were collected along an aragonite saturation range of 0.71-1.11. Samples were grouped in *live*

and of *dead* coral skeletons (skeletons no longer covered in soft tissue) and no difference between the two groups were found. We extend this dataset by including results of 15 *L. pertusa* samples collected from UK waters (three samples from Mingulay Reef, Rockall Bank, Logachev Mound, Pisces 9, and Porcupine Seabight each)[1] which were covered with living tissue at collection. Samples were collected along an aragonite saturation range of 1.67-2.62 and we consider them to be representative of a non-acidified oceanic environment.

Sample preparation was the same as in Hennige et al. [9] and followed previously developed protocols for mineralised tissues[19, 20]. Testing protocol was also kept the same as in Hennige et al. [9]. Briefly, indentations were performed in dry conditions using a Berkovich tip mounted to a depth-sensing, force controlled nanoindenter (Hysitron). Force was applied in a monotonic ramp up to 50 mN over the course of 60 s. Subsequently, force was held constant for 30 s before being unloaded in 7.5 s. 35 indentations per sample (525 indentations in total) were performed and plain strain modulus, hardness, as well as the ratio between elastic and dissipated work were determined following Hennige et al. [9], with plain strain modulus being the interesting variable for validating our polycrystalline modelling. The resulting indentation stiffness is shown in Figure S6c.

References

1. Hennige SJ, Wicks LC, Kamenos NA, Perna G, Findlay HS, Roberts JM. Hidden impacts of ocean acidification to live and dead coral framework. Proc R Soc B 2015, 282(1813)**:** 990-990.
2. Eshelby JD. The Determination of the Elastic Field of an Ellipsoidal Inclusion, and Related Problems. P Roy Soc A 1957, 241**:** 376-396.
3. Mura T. Isotropic Inclusions. In: Evanston IL, editor.: Martinus Nijhoff Publishers; 1987. pp. 74-88.
4. David EC, Zimmerman RW. Compressibility and shear compliance of spheroidal pores: Exact derivation via the Eshelby tensor, and asymptotic expressions in limiting cases. International Journal of Solids and Structures 2011, 48(5)**:** 680-686.
5. Fritsch A, Dormieux L, Hellmich C, Sanahuja J. Mechanical behavior of hydroxyapatite biomaterials: An experimentally validated micromechanical model for elasticity and strength. J Biomed Mater Res A 2009, 88(1)**:** 149-161.
6. Sanahuja J, Dormieux L, Meille S, Hellmich C, Fritsch A. Micromechanical explanation of elasticity and strength of gypsum: from elongated anisotropic crystals to isotropic porous polycrystals. J Eng Mech 2010, 136(2)**:** 239-253.
7. Schwiedrzik JJ, Wolfram U, Zysset PK. A generalized anisotropic quadric yield criterion and its application to bone tissue at multiple length scales. Biomechanics and Modeling in Mechanobiology 2013, 12(6)**:** 1155-1168.
8. Schwiedrzik JJ, Zysset PK. An anisotropic elastic-viscoplastic damage model for bone tissue. Biomech Model Mechan 2013, 12(2)**:** 201-213.

9. Hennige S, Wolfram U, Wickes L, Murray F, Roberts JM, Kamenos N, Schofield S, Groetsch A, Spiesz E, Aubin-Tam ME, Etnoyer P. Crumbling reefs and coral habitat loss in a future ocean. Front Mar Sci 2020, 7**:** 1-16.

10. Green AE, Naghdi PM. A general theory of an elastic-plastic continuum. Arch Ration Mech An 1965, 18(4)**:** 251-281.

11. Perzyna P. The constitutive equations for rate sensitive plastic materials. Q Appl Math 1963, 20**:** 321-332.

12. Etse G, Carosio A. Constitutive equations and numerical approaches in rate depdent material formulations. MECOM 1999.

13. Simo JC, Hughes TJR. *Computational Inelasticity*. Springer New York Berlin et al, 2000.

14. Haugan PM, Evensen G, Johannessen JA, Johannessen OM, Pettersson LH. Modeled and observed mesoscale circulation and wave-current refraction during the 1988 Norwegian Continental Shelf Experiment. Journal of Geophysical Research: Oceans 1991, 96(C6)**:** 10487-10506.

15. Möller T, Trumbore B. Fast, minimum storage ray-triangle intersection. Journal of graphics tools 1997, 2(1)**:** 21–28-21–28.

16. Fantazzini P, Mengoli S, Pasquini L, Bortolotti V, Brizi L, Mariani M, Di Giosia M, Fermani S, Capaccioni B, Caroselli E, Prada F, Zaccanti F, Levy O, Dubinsky Z, Kaandorp JA, Konglerd P, Hammel JU, Dauphin Y, Jean-Pierre Cuif JP, Weaver JC, Fabricius KE, Wagermaier W, Fratzl P, Giuseppe Falini G, Goffredo S. Gains and losses of coral skeletal porosity changes with ocean acidification acclimation. Nat Commun 2015, 6(7785)**:** 1-7.

17. Goffredo S, Mancuso A, Caroselli E, Prada F, Dubinsky Z, Falini G, Levy O, Fantazzini P, Pasquini L. Skeletal mechanical properties of Mediterranean corals along a wide latitudinal gradient. Coral Reefs 2015, 34(1)**:** 121-132.

18. Pasquini L, Molinari A, Fantazzini P, Dauphen Y, Cuif J-P, Levy O, Dubinsky Z, Caroselli E, Prada F, Goffredo S, others. Isotropic microscale mechanical properties of coral skeletons. J R Soc Interf 2015, 12(106)**:** 20150168-20150168.

19. Mirzaali M, Schwiedrzik JJ, Thaiwichai S, Best J, Michler J, Zysset PK, Wolfram U. Mechanical properties of cortical bone and their relationships with age, gender, composition and microindentation properties in the elderly. Bone 2016, 93**:** 196-211.

20. Wolfram U, Wilke HJ, Zysset PK. Rehydration of Vertebral Trabecular Bone: Influences on its Anisotropy, its Stiffness and the Indentation Work with a View to Age, Gender and Vertebral Level. Bone 2010, 46(2)**:** 348-354.
