# Supplementary material for: Multiscale mechanical consequences of ocean acidification for cold-water corals
Source: Sci Rep. 2022 May 16;12:8052. doi: 10.1038/s41598-022-11266-w (PMC9110400; doi:10.1038/s41598-022-11266-w)
Supplement: Supplementary file 5 — Supplementary Legends. [file 41598_2022_11266_MOESM5_ESM.pdf]

Captions to supplementary videos of:

Multiscale Mechanical Consequences of Ocean Acidification for Cold-Water Corals

Uwe Wolfram[1*] Marta Peña Fernández[1] Samuel McPhee[1] Ewan Smith[1] Rainer J. Beck[1] Jonathan D. Shephard[1] Ali Ozel[1] Craig Scott Erskine[1] Janina Büscher[3] Jürgen Titschack[4,5] J Murray Roberts[2] Sebastian Hennige[2]

[1]*School of Engineering and Physical Sciences, Institute of Mechanical, Process and Energy Engineering, Heriot-Watt University, Edinburgh, United Kingdom*
[2]*Changing Oceans Research Group, School of GeoSciences, University of Edinburgh, Edinburgh, United Kingdom*
[3]*GEOMAR Helmholtz Centre for Ocean Research Kiel, Biological Oceanography Research Group, Kiel, Germany*
[4]*Marum Center for Marine Sciences, University of Bremen, Bremen, Germany*
[5]*Senckenberg am Meer, Marine Research Department, Wilhelmshaven, Germany*
[*]*Corresponding author email: u.wolfram@hw.ac.uk*

Video S1 Molecular dynamics simulation of aragonite single crystal tensile strength: These tests were used to determine tensile stiffness and strength of the aragonite crystal as this was not accessible with laboratory experiments. An aragonite volume of interest with $24 \times 6 \times 6$ unit cells of dimension $a \times b \times c$ was used to determine tensile strength of the crystal. Unit cell dimensions are detailed in Section 2.4. The video illustrates a tensile test along the x-axis (Figure 3). Elongation along the x-axis is clearly visible until maximum bearable stress is reached (Figure 3) after which further deformation leads to a reformation of the molecular arrangement. See Section 2.4 for a detailed explanation of the methods and Figure 3 to the colour encoding of the calcium carbonate molecules.

Video S2 Molecular dynamics simulation of aragonite single shear crystal strength: These tests were used to determine shear stiffness and strength of the aragonite crystal as this was not accessible with laboratory experiments. An aragonite volume of interest with $24 \times 24 \times 24$ unit cells of dimension $a \times b \times c$ was used to determine shear strength of the crystal. The video illustrates a shear test in the yz-plane (Figure 3). A simple shear test is performed where the top surface is moved in parallel to the bottom surface. Shear deformation is clearly visible until maximum bearable stress is reached (Figure 3) is reached after which further loading leads to the formation of shear bands (dense regions). Section 2.4 gives a detailed explanation of the methods and Figure 3 to the colour encoding of the calcium carbonate molecules.

Video S3 Visualises incorporation of dissolution on a representative coral specimen: The video illustrates the steps resulting from our image processing shown in Figure 7. The red outline mesh illustrates the original configuration. Underlying grey triangulation illustrates a gradual loss of wall thickness and, at the tips, peripheral mass. Dissolution here follows our measurements of affected layer thickness in Section 4.2 and S2.
